# Nicotinamide Riboside for the Prevention and Treatment of Doxorubicin Cardiomyopathy. Opportunities and Prospects

**DOI:** 10.3390/nu13103435

**Published:** 2021-09-28

**Authors:** Ekaterina Podyacheva, Yana Toropova

**Affiliations:** Research Laboratory of Bioprosthetics and Cardiac Protection, Centre for Experimental Biomodeling, Almazov National Medical Research Centre, Ministry of Health of the Russian Federation, 197341 Saint Petersburg, Russia; yana.toropova@mail.ru

**Keywords:** anthracyclines, doxorubicin cardiomyopathy, NAD^+^ metabolism, nicotinamide riboside, PARPs, sirtuins

## Abstract

Despite the progress in the development of new anticancer strategies, cancer is rapidly spreading around the world and remains one of the most common diseases. For more than 40 years, doxorubicin has been widely used in the treatment of solid and hematological tumors. At the same time, the problem of its cardiotoxicity remains unresolved, despite the high efficiency of this drug. Symptomatic therapy is used as a treatment for side-effects of doxorubicin or pathological conditions that have already appeared in their background. To date, there are no treatment methods for doxorubicin cardiomyopathy as such. A drug such as nicotinamide riboside can play an important role in solving this problem. Nicotinamide riboside is a pyridine nucleoside similar to vitamin B3 that acts as a precursor to NAD^+^. There is no published research on nicotinamide riboside effects on cardiomyopathy, despite the abundance of works devoted to the mechanisms of its effects in various pathologies. The review analyzes information about the effects of nicotinamide riboside on various experimental models of pathologies, its role in the synthesis of NAD^+^, and also considers the possibility and prospects of its use for the prevention of doxorubicin cardiomyopathy.

## 1. Introduction

According to the World Health Organization, cancer is one of the leading causes of death worldwide. Almost 10 million people died from this disease in 2020 [1,2]. Despite the progress in the development of new anticancer strategies, cancer is rapidly spreading around the world and remains one of the most common diseases. At the same time, oncological diseases at late stages are detected in patients in the overwhelming majority of cases. This necessitates chemotherapy as part of a combined therapy and as an independent form of treatment.

For more than 40 years, doxorubicin has been widely used in the treatment of solid and hematological tumors (lymphoblastic leukemia, soft tissue sarcoma, osteosarcoma, breast cancer, thyroid cancer, Wilms tumor, neuroblastoma, bladder cancer, stomach cancer, ovarian cancer, lymphogranulomatosis, non-Hodgkin’s disease lymphomas, trophoblastic tumors, refractory ovarian cancer) [3]. At the same time, the problem of its cardiotoxicity remains unresolved, despite the high efficiency of this drug in ensuring the survival of patients. Cardiotoxicity is dose-dependent and can develop during or immediately after the administration of doxorubicin, as well as some time after the end of treatment. Thus, acute and chronic doxorubicin cardiotoxicity is separated [4,5].

To date, there are no treatment methods for doxorubicin cardiomyopathy, as such. The only drug that is approved by the Food and Drug Administration (FDA) is dexrazoxane. Its action is based on the absorption of free radicals that are generated by iron ions. There are also a number of studies with drugs based on inhibition of angiotensin-converting enzymes (enalapril, zofenopril and lisinopril), beta-blockers (carvedilol) and antioxidants (resveratrol) [3]. Often, symptomatic therapy is used as a treatment for side-effects or pathological conditions that have already appeared in their background, which provides a short-term effect. Therefore, the development of approaches aimed at preventing doxorubicin cardiomyopathy remains relevant. In solving this problem, drugs based on NAD^+^ cofactors, which have a number of interesting properties, can play an important role.

Nicotinamide riboside (NR) is a pyridine nucleoside similar to vitamin B3 that acts as a precursor to nicotinamide adenine dinucleotide (NAD^+^). The exceptional importance of NAD^+^ as a coenzyme is due to its great need for cellular redox reactions, including most catabolic and anabolic reactions, such as glycolysis, fatty acid oxidation, tricarboxylic acid cycle (TCA), synthesis of fatty acids, cholesterol and steroids [6]. Moreover, depletion of NAD^+^ level is promoted by enzymes that consume NAD^+^, such as sirtuins, poly-ADP-ribose polymerases (PARPs), cADP-ribosesynthases (CD38/157 ectoenzymes) and mono-ADP-ribose transferases (ARTs), TIR motif-containing 1 (SARM1) [6,7].

Over the past 10 years, the number of studies devoted to the characteristics of the metabolism of NAD^+^ has increased. This is due to the discovery of nicotinamide riboside kinases. In this regard, researchers from various fields of science are trying to understand the features of nicotinamide riboside and its possible clinical applications. Nicotinamide riboside affects the following pathologies: metabolic syndrome, mitochondrial disorders, DNA repair syndromes, Alzheimer’s disease, fatty liver disease, hepatic carcinoma, inflammatory conditions, cardiomyopathy, noise induced hearing loss, aging and diseases of aging [8].

Data on the biochemical aspects of NR are the basis for it to be considered as a pathogenetically valid therapeutic agent for the prevention or treatment of doxorubicin cardiomyopathy. Despite the abundance of work devoted to the mechanisms of realization of the effects of NR in various pathologies, there has been no such work in cardiomyopathy. This review aims to fill this gap. The article analyzes information about the effects of NR on various experimental models of pathologies, its role in the synthesis of NAD^+^, and also considers the possibility and prospects of its use for the prevention of doxorubicin cardiomyopathy.

## 2. Article Search and Selection Strategy

The search for published articles and reviews in peer-reviewed open access journals was carried out using the databases PubMed and Google Scholar. In addition, we used existing abstracts of articles or entire articles on ResearchGate without open access. Most of the peer-reviewed articles were published within the past 15–20 years. Older work was seen rather as a source of fundamental discoveries. Additional databases were also searched through Google using the following keywords: anthracycline cardiotoxicity, doxorubicin, nicotinamide riboside, nicotinamide riboside bioavailability, nicotinamide riboside safety, NAD^+^ metabolism, NAD^+^, Sirtuins, and PARP.

## 3. Features of the Development of Doxorubicin Cardiomypathy

The mechanisms of anthracycline cardiotoxicity are still being investigated in various experimental models [5]. All anthracyclines (doxorubicin, daunorubicin, epirubicin, idarubicin) have a general mode of ultrastructural myocardial damage, which is characterized by loss of myofibrils, expansion of the sarcoplasmic reticulum, cytoplasmic vacuolization, swelling of mitochondria and an increase in the number of lysosomes [9]. At this point in time, there are two main hypotheses for the development of doxorubicin cardiomypathy.

The first hypothesis is based on the formation of a stable ternary complex anthracyclin-DNA-topoisomerase IIβ. DNA topoisomerases induce transient single-or double-stranded breaks that regulate topological changes during DNA replication, transcription, recombination, and chromatin remodeling. In humans, TopII is presented in the form of TopIIα and TopIIβ isoenzymes [10]. TopIIα is highly expressed in malignant and benign (highly proliferating) cells. In contrast, TopIIβ is highly expressed in resting cells, such as adult mammalian cardiomyocytes. Anthracyclines can easily penetrate cells and localize in the nucleus [11]. Doxorubicin binds to DNA and topoisomerase IIβ, thus forming the anthracycline-DNA-topoisomerase IIbeta ternary complex, which, in turn, causes double-stranded DNA breaks. When bound to TopIIα, the complex inhibits DNA replication, stops the cell cycle, and induces apoptosis [12]. This is how doxorubicin works in proliferating malignant cells. Conversely, when the complex binds to TopIIβ in cardiomyocytes, it leads to the activation of an altered P53 tumor suppressor pathway, β-adrenergic signaling, mitochondrial dysfunction, impaired calcium processing and increased necrosis/apoptosis [11]. Thus, anthracyclines “poison” the enzyme, prevent repeated ligation of double-stranded DNA breaks and initiate programmed cell death [11,13]. This theory is supported by data from experimental studies using knockout mice. Top2β (KO) knockout mice showed that lack of Top2β protects against doxorubicin-induced cardiotoxicity [14], partly by reducing mitochondrial dysfunction.

**Figure 1:**
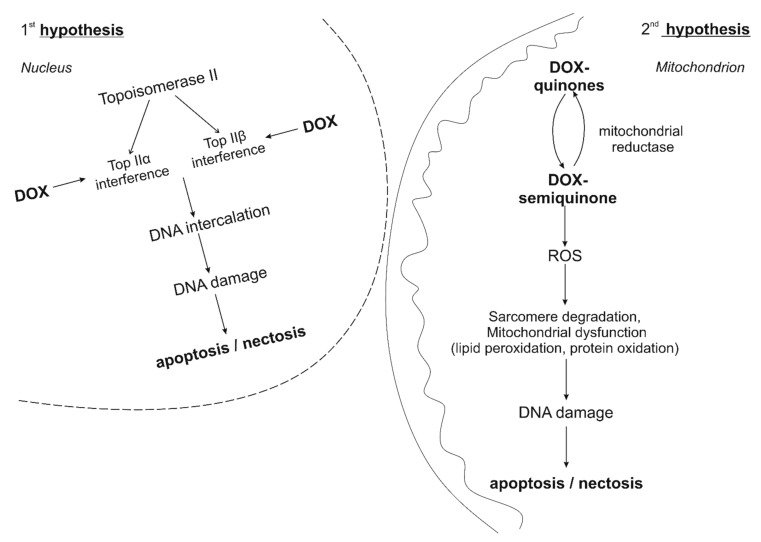
Features of the development of doxorubicin cardiomypathy.

The second hypothesis is that doxorubicin-induced cardiomyopathy is closely associated with an increase in oxidative stress, as evidenced by reactive oxygen species (ROS)-induced damage such as lipid peroxidation, as well as decreased levels of antioxidants and sulfhydryl groups. The quinone fragment of anthracyclines can undergo one-electron reduction by reductases localized in mitochondria to form semiquinone. Further, the semiquinone oxidized in this way exposes the cell to ROS levels higher than physiological levels (for example, superoxide anion, hydrogen peroxide and hydroxyl radical) [15]. The reaction of superoxide anion with nitric oxide also generates peroxynitrite, endowed with prooxidant activity [15]. It is known that cardiomyocytes are very rich in mitochondria, in comparison with other types of cells, but relatively poor in enzymes that can resist the increasing amount of ROS. Consequently, cardiomyocytes are easily susceptible to sustained increased ROS generation, which causes sarcomere degradation, mitochondrial dysfunction and DNA damage, ultimately leading to disruption of the expression of heart-specific genes, apoptosis/necrosis [16,17,18]. This is the second popular “oxidative stress” hypothesis of cardiotoxicity. A decrease in biochemical and functional parameters of cardiotoxicity was observed in cell, mouse and rat models with the addition of natural antioxidants, probucol (originally developed as a drug that reduces lipid) and dexrazoxane [13,15,19]. The “oxidative stress” hypothesis of cardiotoxicity was also successfully investigated by Kang et al. in 1996 on transgenic mice that overexpressed antioxidant defense systems such as catalase [20], and investigated by Yen et al. in 1997 on mitochondrial manganese-dependent superoxide dismutase [21] The features of the development of doxorubicin cardiomypathy are summarized in Figure 1.

Therefore, the existing theories of the development of doxorubicin-induced cardiomypathy imply the involvement of oxidative stress, which provides the basis for the potential effectiveness of pharmacological action, providing an effect on this link.

## 4. Known Pathways of the Synthesis of NAD^+^ Feature of NR

Bieganowski and Brenner in 2004 first described the direct contribution of NR to the metabolism of NAD^+^ [22]. In this study, the authors characterized NR kinase enzymes (NRKs), that are capable of converting NR directly to nicotinamide mononucleotide (NMN), bypassing nicotinamide phosphoribosyltransferase (NAMPT) in the salvage pathway of NAD^+^ synthesis. Since the reaction catalyzed by NAMPT limits the rate, it requires the use of energy-consuming PRPP (5-phosphoribosyl 1-pyrophosphate) and must be inhibited by NAD^+^. NR is also able to increase the concentration of NAD^+^ beyond what is achieved through normal metabolism of vitamin B [6,23].

**Figure 2:**
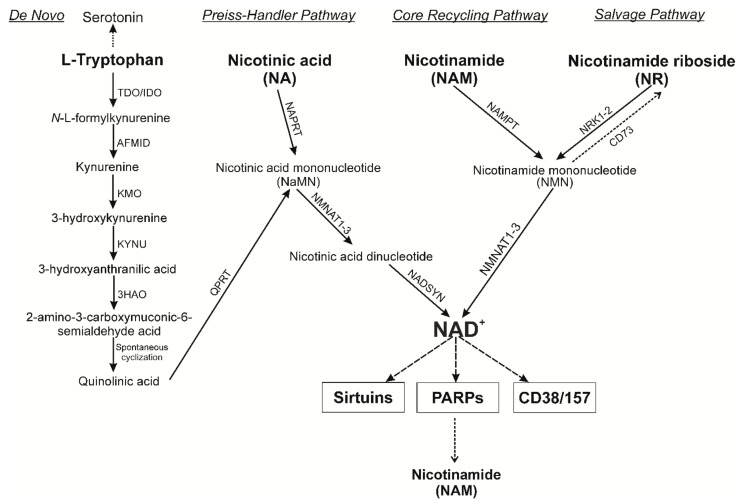
NAD^+^ biosynthetic pathways. *De novo* biosynthesis begins with the conversion of tryptophan (Trp) to *N*-L-formylkynurenine either by indolamine-2,3-dioxygenase (IDO) or tryptophan-2,3-dioxygenase (TDO). After four reaction steps, *N*-L-formylkynurenine can subsequently be converted to the unstable 2-amino-3-carboxymuconate-6-semialdehyde acid, which can undergo nonenzymatic cyclization to quinolinic acid. The last step of de novo biosynthesis consists of the quinolinate-catalyzed phosphoribosyltransferase (QPRT) formation of NA mononucleotide (NaMN) using PRPP (5-phosphoribosyl 1-pyrophosphate). *The Preiss-Handler pathway* is initiated by NA phosphoribosyltransferase (NAPRT) to form NaMN. Further, NAMN is converted into adenine dinucleotide NA (NAAD) by the enzymes NMN adenylyltransferase (NMNAT1-3) together with ATP. Finally, NAAD is converted to NAD^+^ via an amidation reaction catalyzed by the enzyme NAD^+^ synthase (NADSYN). The *Core Recycling* and *Salvage Pathways* are the shortest ways to synthesize NAD^+^ from NAM and NR (2 steps). NAM is converted by the rate-limiting nicotinamide phosphoribosyltransferase (NAMPT) to form NMN using PRPP as a cosubstrate. NMN is also a product of NR phosphorylation by NR kinases (NRK1-2). The subsequent conversion of NMN to NAD^+^ is catalyzed by the NMNAT1-3 enzymes. Further, the synthesized NAD^+^ is used in the work of such enzymes as sirtuins, PARPs, CD38/157. NR, nicotinamide riboside; NAM, nicotinamide; NMN, NAM mononucleotide; AFMID, kynurenine formamidase; KMO, kynurenine 3-monooxygenase; KYNU, tryptophan 2,3-dioxygenase; 3HAO, 3-hydroxyanthranilic acid oxygenase.

It is important to note that due to the central role of NAD^+^ in cellular bioenergetics and the maintenance of relatively high concentrations of NAD^+^ metabolites in cells (usually 200–500 μM in mammalian cells), several different pathways are involved in the biosynthesis of NAD^+^ [24]. In humans, it includes the eight-step *de novo* pathway from the amino acid tryptophan precursor and additional three and two-step pathways from various nicotinoyl precursors such as nicotinic acid (NA) and nicotinamide (NAM), as well as nucleosides, NR, and nicotinic acid riboside (NAR). In general, NAD^+^ metabolism can be divided into four main categories: *de Novo* synthesis; *the Preiss-Handler pathway, the Salvage pathway* from the corresponding precursors NA, NR and NAR, and the *Core Recycling Pathway* via Nicotinamide [8,24,25]. In mammals, the most common precursor is NAM, which can then be used to generate NMN by the rate-limiting enzyme NAMPT [26]. Finally, NMN is converted to NAD^+^ by NMN/NaMN adenylyltransferases (NMNAT). It was found that the expression of NAMPT and, accordingly, the content of NAD^+,^ decreases in many tissues depending on the aging process, overeating, stress or inflammatory factors of various origins [27]. In this regard, an important role is played by the fact that the maintenance of NAD^+^ levels depends on different biosynthetic pathways and precursors in different tissues [28,29,30,31]. Nevertheless, a decrease in the expression of the NAMPT enzyme is one of the main reasons for a pronounced decrease in the NAD^+^ level (Figure 2).

The need for NAMPT can be circumvented by direct conversion of NR to NMN by two nicotinamide ribosokinases, NRK1 and NRK2 [32]. Therefore, we will pay special attention to the synthesis of NAD^+^ from nucleosides (NR and NAR). NRK1 and NRK2 are encoded by the human genome and by the genomes of other mammalian organisms (encoded by the Nmrk1 and Nmrk2 genes, respectively) [32,33]. Synthesis of NMN with NRK avoids the need for energy-intensive PRPP. Additionally, NR can be converted to NAM by purine nucleoside phosphorylase (NP), which is subsequently converted to NAD^+^ via NMN synthesis by NMNAT [30,34,35]. NR and NAR increase NAD^+^ levels dose-dependently and up to 2.7-fold in a single 1000 mg dose in mammalian cells, in contrast to nicotinamide or nicotinic acid at the same concentrations [34,36,37]. Kulikova et al. in 2015 showed that NR can be produced in mammalian cells and released extracellularly, suggesting possible intercellular metabolic networks involving the creation, release and transport of NR (and NAR) to other cells [38].

The effectiveness of NR in increasing cellular NAD^+^ has led to studies questioning whether it can treat diseases such as cardiovascular complications of various origins, neurodegenerative disorders or metabolic syndromes [8], based on the idea that a decrease in NAD^+^ levels may be a risk factor in these states.

## 5. Metabolism of NAD^+^

The importance of NAD^+^ is reflected in its balance in various cellular compartments, and this balance is manifested in the activity of enzymes that consume NAD+ (sirtuins, PARPs, CD38/157, SARM1), stress and aging mediators, the production of which is enhanced by factors such as DNA damage, oxidative stress and inflammation. The sirtuin family (NAD^+^-dependent deacetylases/deacylases) includes seven genes, and the corresponding proteins encoded by them, with different localization within the cell, enzymatic activity and targets (mitochondrial SIRT3-5; nuclear SIRT1,6,7; cytoplasmic SIRT2; SIRT1 is able to move between the nucleus and the cytoplasm) [39,40]. Sirtuins are conservative regulators of aging and longevity in various organisms and are considered major metabolic switches due to their many regulatory functions in metabolism, DNA repair, stress response, chromatin remodeling, and circadian rhythm. SIRT1 and SIRT2 are believed to be responsible for most NAD^+^ consumption under normal conditions. Increased NAD^+^ levels strongly correlate with sirtuin activation during fasting and calorie restriction [41]. The enzymatic mechanisms of sirtuins in the regulation of cellular metabolism are still being actively studied. At this stage, it is known that the enzymatic activity of sirtuins includes the removal of the acetyl group from the lysine residues of the target proteins in a two-step process. First, NAD^+^ is hydrolyzed to NAM and ADP-ribose, then the acetyl group of the target protein is cleaved and transferred to ADP-ribose, providing the formation of an intermediate peptidyl-ADP-ribose. Acetyl-ADP-ribose is subsequently released. Previously, it was impossible to investigate changes in NAD^+^ levels and sirtuin activity due to technical difficulties. It is now known that nuclear SIRT1, SIRT6 and SIRT7 are critical regulators of DNA repair and genome stability; mitochondrial SIRT3, SIRT4, SIRT5 and nuclear SIRT1 regulate mitochondrial homeostasis and metabolism. SIRT1 also plays a significant role in the turnover of defective mitochondria, thus being a key factor in maintaining the quality of mitochondrial metabolism [42,43,44,45]. Canto in 2012 described a number of transcription factors that can regulate the expression of SIRT1 under starvation conditions: CREB, PPARs, FOXOs, and p53. Conversely, ChREBP transcription factors activated by high glucose uptake suppress SIRT1 levels [46]. In turn, SIRT2 targets key metabolic regulators such as FOXO, the p65 NF-kB subunit, and phosphoenolpyruvate carboxykinase (PEPCK) [41,47,48], suggesting its role in the regulation of inflammation, gluconeogenesis, and the response to caloric restriction. SIRT3 target proteins include mitochondrial respiratory complexes, TCA cycle proteins, and enzymes associated with lipid metabolism and detoxification of reactive oxygen intermediates, such as isocitrate dehydrogenase (IDH) and superoxide dismutase (SOD). PGC-1α, as the main organizer of mitochondrial biogenesis, positively regulates SIRT3 at the transcriptional level in response to various energy stresses and starvation [49]. SIRT4 and 5 are currently less studied but SIRT4 is known to act as a modulator of fat metabolism in hepatocytes and myocytes. SIRT4 contributes to the opposite effects of SIRT1 on insulin secretion [50], and SIRT3 contributes to the opposite effects of SIRT1 on fat oxidation [51]. Du in 2011 discovered that the main function of SIRT5 is not as a deacetylase but as a demalonizase and desuccinylase [52]. SIRT6 is attracting research for its role in genomic DNA stability, metabolism, and aging. Its pronounced expression provides protection against obesity associated with a high-fat diet, and SIRT6 is also able to work as a corepressor of HIF-1alpha [53]. SIRT6 knockout mice demonstrate severe defects such as lymphopenia, loss of subcutaneous fat, decreased bone mineral density, hypoglycemia, and decreased levels of insulin-like growth factor (IGF)-1. SIRT7 is localized in the nucleolus and has been described as a component of the transcriptional apparatus of RNA polymerase I (Pol I) [54]. There is also evidence of a possible role for SIRT7 in cancer development, but further study is required [55]. In general, we could say that at this stage of the development of science, sirtuins are firmly established and have become central participants in understanding how NAD^+^ levels affect cellular homeostasis and how exactly, in the future, they may be used in a therapeutic direction.

The PARP family of proteins also use NAD^+^ in large quantities in their work, together with sirtuins. The human PARP family includes 17 proteins characterized by poly (ADP-ribosyl) polymerase activity or mono (ADP-ribosyl) polymerase activity. Ones catalyze the reaction of transfer of ADP-ribosyl (adenosine diphosphate-ribose residue) to a poly-ADP-ribosyl chain bound to a protein, in which the donor of ADP-ribose is NAD^+^. It is known that only PARP1, PARP2, and PARP3 are localized in the nucleus, respond to DNA breaks, and contribute to the DNA repair process [56]. PARP activation increases as DNA damage accumulates over time, while SIRT1 activity decreases due substrate competition, being in the same cellular compartment, i.e., the nucleus. It is also important to note that PARP1 has higher binding affinity and faster kinetics for NAD^+^ compared to SIRT1 [40]. To date, PARP1 is the best characterized of all members of the family. This molecule is widely associated not only with the aging process due to its high activity of NAD^+^ consumption during DNA repair, but also with other normal and pathophysiological processes, confirming its key role in maintaining homeostasis in the cell [56]. For example, there is a strong correlation between PARP activation, decreased SIRT1 activity, and decreased NAD^+^ levels in patients with group A xeroderma pigmentosa, ataxia, telangiectasia, and Cockayne’s syndrome [57]. Treating cocaine-treated mice with NAD^+^ precursor supplementation has been shown to increase lifespan and reduce severe phenotypic manifestations caused by PARP1 hyperactivation, providing strong evidence that the negative consequences of PARP1 activation are mediated by the dysregulation of NAD^+^ homeostasis in response to extensive DNA damage and genotoxic stress [58]. As for the rest of the members of the PARP family, PARP2 and PARP3 are structurally related to PARP1, and have a similar catalytic domain required for the regulation of DNA repair and transcription [59,60]. The functions and effects of PARP4-7 have not yet been definitively determined on metabolism of NAD^+^.

In addition, ectoenzymes CD38/157 with glycohydrolase and ADP-ribosyl cyclase activity, use NAD^+^ for the production of cADP-ribose and NAAD(P) (nicotinic acid adenine dinucleotide (phosphate)). In turn, cADP-ribose and NAAD(P) are secondary messengers that promote the mobilization of Ca^2+^ [61]. This fact determines the understanding of the role of CD38/157 in modulation of many cellular processes such as survival, metabolism, and activation of immune cells, and also in the biology of aging, for example, age-related diseases (rheumatoid arthritis and cancer) [62]. CD38 can also degrade NAD^+^, NR and NMN intermediates, which further reduces NAD^+^. The effect of CD38 on NAD^+^ content has been demonstrated in CD38-deficient mice in which NAD^+^ levels remain high [63]. This preserves mitochondrial respiration and metabolic function with age. Moreover, inhibition of CD38 can increase NAD^+^ levels and improve glucose and lipid metabolism [63,64]. Although CD38 and CD157 are members of the same enzymatic family and are genetically homologous, they are structurally and localized differently. CD38 is a type II or type III transmembrane protein, first described in the late 1970s as a marker of T cell activation. CD38 is now known to be ubiquitous, especially during inflammation [65]. CD157 is a glycophosphatidylinositol-anchored protein that was first identified in the myeloid compartment of the hematopoietic system. It is also expressed by other cells, including B-cell progenitors, Paneth cells, and endothelial cells in the intestine, pancreas, and kidneys [66]. In addition to enzymatic function, CD38 and CD157 also work as cellular receptors. Deaglio et al. indicated a role for CD38 as an adhesion receptor that interacts with CD31 to mediate the transport of immune cells and their movement through the endothelium. Thus, it activates the proliferative response in lymphocytes of chronic lymphocytic leukemia, confirming the detrimental role of CD38 in blood cancer [67,68]. Still, CD157 as a receptor remains poorly understood.

**Figure 3:**
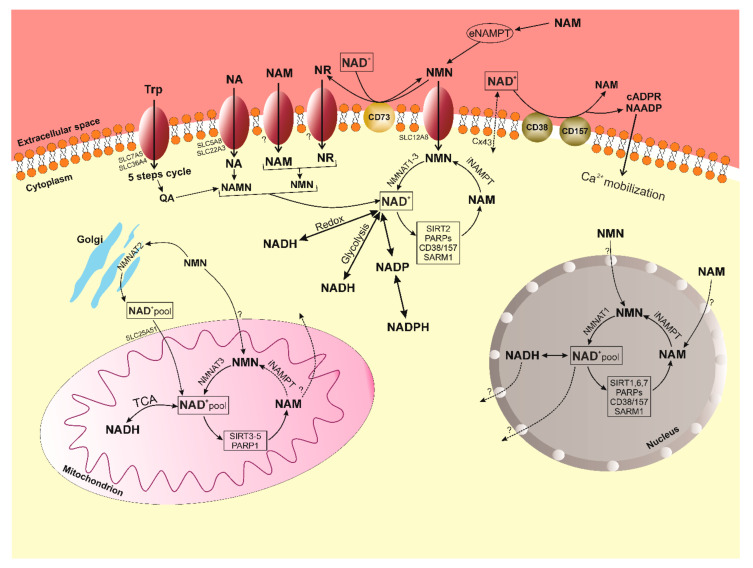
The balance of NAD^+^ is the balance of synthesis, consumption and recirculation in various subcellular compartments (cytoplasm, nucleus and mitochondria). After entering the cell, NAD^+^ precursors are metabolized by four main pathways (Figure 1) to NAD^+^. In the cytosol, nicotinamide (NAM) is converted to nicotinamide mononucleotide (NMN) by the intracellular form of NAM phosphoribosyltransferase (iNAMPT). NMN is then converted to NAD^+^ by NMN transferase 2 (NMNAT2) bound to the outer Golgi membrane in the cytoplasm. NAD^+^ is converted to NADH/NADPH during the redox cycle, used during aerobic/anaerobic glycolysis and is consumed by NAD-dependent enzymes (SIRT2, PARP1-3, CD38/157, SARM1). In mitochondria, NMN is converted to NAD^+^ by NMNAT3. NAD^+^ is used by the TCA cycle to generate ATP and is additionally used by mitochondrial sirtuins 3–5 (SIRT3-5) and PARP1, which generate NAM. Studies show the presence of NAD^+^, NADH and NMN transporters in the mitochondrial membrane, but no specific transporters have yet been identified [27]. It is still not clear whether NAM can be converted back to NMN within the mitochondria, or whether it is transported/diffused from the mitochondria into the cytosol [39]. Inside the nucleus, NMN is converted to NAD^+^ by NMNAT1, while NAD^+^ is consumed here mainly by SIRT1,6,7, PARP1-3, SARM1. As in the cytosol, NAM is returned back to the NMN by iNAMPT.

SARM1 is the new enzyme involved in the metabolic reactions of NAD^+^. SARM1 is an enzyme that is the most evolutionarily conserved member of the Toll-interleukin receptor (TIR) family. SARM1 is able to hydrolyze NAD^+^ into cADPR and therefore functions as a Ca^2+^ signaling enzyme similar to CD38; however, SARM1 increases cADPR much more efficiently than CD38 [69]. It also plays a key role in the degeneration of axons after damage. It is known to be more expressed in neurons and promotes neuronal morphogenesis and inflammation [69]. SARM1 triggers an axonal destruction program that catalyzes the production of nicotinamide and ADPR/cADPR from NAD^+^, causing bioenergetic depletion of NAD^+^ and ATP in response to neuronal damage. This is followed by the activation of calpain and the final dismantling of the axon [70]. Additionally, SARM may play a role in mitophagy and possibly other, as yet unknown, cellular functions, but its main known function in mammals is to mediate neuronal cell death [71]. Key points of NAD^+^ metabolism are presented in Figure 3.

The role of NAD^+^ as a coenzyme in most metabolic pathways suggests that NAD^+^ limitations affect metabolic efficiency and, therefore, NAD^+^ levels may change during various physiological processes. This is confirmed by a number of studies on worms, rodents and human cell models [72,73,74]. For instance, a decrease in NAD^+^ content in muscle progenitor cells leads to a SIRT1-mediated metabolic switch that induces premature differentiation and loss of regenerative capacity, reflecting a phenotype typical of aging muscles [75]. The link between metabolism and NAD+ is further supported by the observation that tissue NAD^+^ levels are reduced with diets high in fat [44]. In contrast, NAD^+^ increases in response to exercise or calorie restriction [43,76]. The addition of NAD^+^ precursors has been shown to increase the lifespan of budding yeast and worms [45]. In the studies of Khan et al. and Cerutti et al. in 2014 in mammals, an increase in NAD^+^ levels was associated with an improvement in mitochondrial function under stress conditions, which in turn led to protection against metabolic complications of various origins [77,78]. It is also important to note that liver NAD^+^ levels are dynamically altered by circadian rhythms. The heterodimeric complex of the main factors of circadian transcription BMAL1 and CLOCK controls the expression of the *Nampt* gene encoding the NAMPT enzyme. The activation of circadian transcription factors decreases under the influence of various inflammatory cytokines and oxidative stress; therefore, the synthesis of NAD^+^ is impaired. Circadian transcription factors are controlled by SIRT1 according to the principle of feedback, which also regulates the expression of the *Bmal1* and *Clock* genes in the suprachiasmotic nucleus through the RORα and PGC-1α complex [79,80]. This important aspect also demonstrates the ability of NAD^+^ to dynamically respond to various physiological stimuli.

Since glycolysis in the cytoplasm, and the TCA cycle in mitochondria, are able to influence metabolic homeostasis by changing the cytosolic and nuclear levels of NAD^+^/NADH, the concentration of NAD^+^ is always limited [79,81]. After any DNA damage, NAD^+^ levels can drop so low that glycolysis and the flow of substrate into mitochondria are blocked, eventually leading to cell death [30,39,82]. This fact emphasizes the need to understand the mechanisms of NAD^+^ metabolism, and the relationship of its precursors, since their homeostasis and interaction are important for maintaining cell viability and ATP levels. It is also important to understand how exactly it can be used as a possible therapeutic agent for the prevention of various pathologies/complications; for example, doxorubicin cardiomyopathy.

## 6. Role of NAD+/SIRT in the Regulation of Oxidative Stress

As mentioned above, the generation of ROS plays a huge role in the development of doxorubicin cardiomyopathy. It has been demonstrated that NAD+ and SIRT1, through deacetylation of various substrates, regulate important metabolic processes, including oxidative stress and apoptosis [83]. It is also known that inhibition of SIRT1 by pharmacological agents can lead to an increase in ROS levels. This fact indicates a certain relationship between SIRT1 and ROS [84]. SIRT1 has received a lot of attention due to its role in resistance to oxidative stress. The currently known mechanisms include the SIRT1/FOXOs, SIRT1/NF-κB, SIRT1/SOD, SIRT1/NOX.

It was shown that Sirt1 and FOXO3 are capable of forming a complex both in vivo and in vitro, stimulated by oxidative stress. SIRT1 is able to deacetylate FOXO3 to induce resistance to oxidative stress [83]. Similarly, SIRT1 binds to FOXO1 by an NAD-dependent pathway and allows the accumulation of FOXO4 in the nucleus by producing DNA damage-induced protein 45 via a gene associated with stress resistance [85]. Thus, the interaction between SIRT1 and FOXO in response to oxidative stress enhances antioxidant effects and prevents endothelial dysfunction [86]. Liu et al. in 2015 showed that SIRT1-dependent activation of FOXO1 is critical for vascular protection after the beginning of oxidative stress [87].

It has been shown that the activation of NF-κB is closely related to the generation of ROS. Antioxidant genes are controlled by NF-κB [83]. NF-κB and SIRT1 can participate in antagonistic relationships during ROS regulation. SIRT1 inhibits the NF-κB pathway and naturally suppresses ROS production by deacylating p65 subunits or activating AMPK and PPARα. In addition, NF-κB transcription suppresses SIRT1 activation through ROS production [88]. Thus, SIRT1 regulates NF-κB signaling and controls the increase in ROS. At the same time, NF-κB can reduce SIRT1 levels to increase ROS production.

Superoxide dismutase (SOD) is known to play a key role in resistance to oxidative stress. Deficiency or damage of metals in SOD, including Cu-SOD, Zn-SOD, Ni-SOD, Mn-SOD, and Fe-SOD, directly contributes to oxidative stress [89]. Shimada et al. showed that SIRT1 promotes the expression of manganese superoxide dismutase (MnSOD) and thus increases resistance to oxidative stress in endothelial cells of human retinal microvessels [90]. SIRT1 also enhances FOXO3a activation by deacetylation, which increases the transcription of genes such as MnSOD. Therefore, SIRT1/FOXO/MnSOD may promote resistance to oxidative stress in endothelial cells [83]. Moreover, an increase in NAD^+^ levels, SIRT1 expression and an increase in MnSOD were found after NMN treatment in the thoracic aorta [91].

Sirt1 is involved in regulating the production of NADPH oxidase (NOX). It has been shown that Sirt1 is a key participant in cellular senescence and depends on NAD^+^. Decrease in NAD^+^ content caused by ROS tends to decrease SIRT1 activity [83]. However, increased NOX activity can increase NAD^+^ and SIRT1 levels, causing an oxidized state in endothelial cells. These effects are explained by a small and temporary increase in ROS, which induces the expression of SIRT1 [92].

## 7. Effects of NR in Small Laboratory Animal Models

The study of Canto et al. in 2012 is the first major study of the effect of NR on the metabolic state of C57Bl/6J mice following a high-fat diet. In Canto’s study, mice fed 400 mg/kg /day of NR in the diet were protected from weight gain, were more sensitive to insulin, and had increased mitochondrial content in skeletal muscle and brown adipose tissue compared to untreated controls [93].

A study in 2014 by Kevin D. Brown showed that a 1000 mg/kg NR injection given to mice twice daily for 5 days prevented noise-induced hearing loss (NIHL) and spiral ganglion neurite degeneration, even after exposure to noise [94]. Brown demonstrated that these effects were mediated by a NAD^+^-dependent mitochondrial sirtuin, SIRT3. Since mice overexpressing SIRT3 are resistant to NIHL, deletion of SIRT3 reverses the protective effects of NR and the expression of biosynthetic NAD^+^ enzymes. These data indicate that NR administration activates the NAD^+^-SIRT3 pathway, which reduces noise-induced neurite degeneration. NR has a therapeutic effect in various muscle pathologies. For instance, NR improves mitochondrial function, induces autophagy in mitochondrial myopathies, and decreases the mitochondrial unfolded protein response in a model of heart failure caused by cardiospecific transferrin receptor deletion [77]. Cerutti et al. used a diet (400 mg/kg HP) for four weeks. Frederick et al. in 2016 showed that injection of NR to both female and male mice eliminates progressive wasting syndrome in the Duchenne muscular dystrophy model and restores animal endurance with one week of treatment by adding 400 mg/kg NR to drinking water [95] NR generally increases lifespan and is thought to be based on improved stem cell function [96]. Gong et al. demonstrated dramatic improvement in Alzheimer’s disease in a Tg2576 mouse model with a diet of 250 mg/kg NR in a 2013 study [6,97].

The studies of Trammell et al. are of particular interest. They determined the dose-dependent effects of NR on the metabolism of NAD^+^ in human blood [35,98,99,100]. The authors showed that a single oral dose of 1000 mg of NR in humans can increase blood NAD^+^ levels 2.7 times, and that oral administration of NR increases liver NAD^+^ levels in mice with excellent pharmacokinetics superior to those of nicotinic acid and nicotinamide. Separately, Trammell investigated single doses of 100, 300, 1000 mg NR in humans [35]. He also worked with C57Bl/6J mice and injected intraperitoneally (IP) 500 mg/kg NR for 6 days [100].

NR administration prior to sepsis simulation prevents lung and heart damage and improves survival in mice by inhibiting oxidative stress through NAD^+^/SIRT1 signaling and HMGB1 (plasma high mobility group box-1) release [101]. Honga et al., in 2018, injected 100, 300, 500 mg/kg NR intraperitoneally into C57Bl/6J mice 30 min before the injection of feces into the peritoneum [101]. Zheng et al. in 2019 also administered 100, 300, and 500 mg/kg NR in a single dose of IP 30 min before injection of 20 mg/kg doxorubicin [102]. This study showed that NR administration increased NAD^+^ levels and decreased heart damage and myocardial dysfunction in chemically-treated mice. Similar protective effects of NR have been replicated in cultured cardiomyocytes after doxorubicin treatment. NR prevents blockage of autophagic flow, accumulation of autolysosomes, and oxidative stress in cardiomyocytes. In general, NR increases the clearance of autolysosomes through NAD^+^/SIRT1 signaling, thereby preventing doxorubicin cardiotoxicity [102]. There are also studies demonstrating the positive effect of NR on the models of retinal degeneration in BALB/c mice, which were injected with IP 1000 mg/kg of the drug [103].

Based on the existing information to date, it can be concluded that researchers, since the beginning of the study of NR as a precursor of NAD^+^ metabolism, have focused their attention on the introduction of the drug from 100 to 1000 mg/kg. Each has chosen their dose of NR based on previous work and the needs of their research, using two methods of drug administration: oral and intraperitoneal.

Conze and his colleagues conducted a large study to determine the safety of the synthetic analogue of NR from Niagen^TM^ using the reverse mutagenesis assay of bacteria (Ames assay), the analysis of chromosomal aberrations in vitro, and the analysis of micronuclei in vivo, and also investigated the toxicity of the drug in male and female Sprague-Dawley rats within 14 and 90 days [104]. Initially, they carried out work on the study of acute toxicity after oral administration of 5000 μ/kg NR. There was no mortality from such a dose. Further, based on the results of a 14-day study with daily administration of NR at 750, 1500, 2500, 5000 mg/kg, work was carried out to study the subchronic toxicity of Niagen, which lasted 90 days. Animals received 300, 1000, 3000 mg/kg of the drug orally. The results of the study demonstrated that NR is not genotoxic and that the toxicity profile of NR is similar to that of nicotinamide at the highest dose tested. The lowest level of side effects was observed at a dose of 1000 mg/kg, and toxicity was completely absent with the introduction of 300 mg/kg. The main target organs were the liver, kidneys, ovaries and testes [104].

Based on the study of Conze et al. in 2016, a protocol for oral administration of HP was selected in a number of studies by Kourtzidis et al. in 2016 and 2018. They studied the effect of the NAD^+^ precursor on physical performance by daily oral administration of 300 mg/kg NR to male Wistar rats for 21 days [105,106]. They showed that chronic NR intake increases NADPH levels, dramatically increases liver glycogen but not muscle glycogen, decreases antioxidant enzyme activity, decreases blood glucose levels, and maximizes lactate production during exercise. Kourtzidis et al. indicated that long-term intake of NR can lead to dysregulation of redox and energy metabolism and impairment of physical health in rats [105,106]. Thus, an exogenously administered drug to healthy people can lead to undesirable side effects in addition to positive effects.

Hamity et al. in 2017 showed in female Sprague-Dawley rats that were orally administered 200 mg/kg NR before intravenous injection of paclitaxel (a model of paclitaxel-induced peripheral neuropathy) and after 24 days, a decrease in the development of tactile sensitivity and dulling of avoidance behavior places [107]. Hamity’s results suggest that agents that increase NAD^+^ are key cofactors for mitochondrial oxidative phosphorylation systems and cellular redox systems involved in energy metabolism, and represent a novel therapeutic approach for the relief of chemotherapy-induced peripheral neuropathies.

In 2018, a study was published that used a completely new method of introducing NR [108]. This study investigated the effect of NR by intravenous administration of 50 mg/kg, on the functional state of the endothelium, microcirculation and intestinal morphology in acute mesenteric ischemia and reperfusion. The results showed that NR improves the relaxation function of mesenteric vessels and contributes to the protection of the intestinal wall from ischemia-reperfusion injury [108].

An interesting study was carried out in 2020 by de Castro et al., that demonstrated the possibility of reducing oxidative stress in the myocardium of Wistar rats in an obesity model [57].

There have been few studies done on rats to date. Researchers have often used only the oral route of HP administration and have focused their attention on the introduction of 200–300 mg/kg, referring to the article by Conze, Crespo-Barreto and Kruger, 2016. Table 1 summarizes the data on the studies described in this section.

## 8. Supposed Role of NR in the Prevention of Doxorubicin Cardiomyopathy

NAD^+^ metabolism plays a key role in the regulation of cell life (glycolysis, fermentation, pyruvate dehydrogenase, TCA cycle and oxidative phosphorylation) due to the fact that NAD^+^ levels are important for optimizing metabolic parameters in both normal and pathological conditions. It is important that NAD^+^(H) levels are in a constant balance between synthesis and consumption in various cellular compartments, such as the nucleus, mitochondria and cytoplasm, to maintain redox homeostasis. Deficiency of various etiologies leads to redox stress and is accompanied by the development of pathological conditions. An example of this development is the action of anthracycline antibiotics.

To date, as mentioned above, the mechanisms of the damaging effect of doxorubicin on the myocardium, and possible ways of preventing them, are being actively studied. The mechanisms of action of doxorubicin are based on its intercalation with DNA and inhibition of topoisomerase IIβ. The cardiotoxicity of doxorubicin depends on various signaling mechanisms. Above all, doxorubicin-induced cardiotoxicity is caused by the development of oxidative stress. Doxorubicin undergoes a redox cycle in complex I of the electron transport chain, which leads to massive production of ROS and subsequent damage to DNA, proteins and lipids, ultimately leading to cell dysfunction and cell death.

The balance between the formation of free radicals and antioxidant defense systems is disturbed in diseases associated with oxidative stress. Free radicals in large quantities damage lipids, DNA and proteins, and can react with metal bound to proteins, affecting all vital components of cells and tissues. Various enzyme systems are designed to deal with free radical damage and thereby protect against free radical-induced diseases, among which PARPs and SIRTs that consume NAD^+^ may play a key role. However, it is important to understand exactly how ROS cause DNA damage, and therefore activate PARP, in an attempt to restore DNA integrity. PARP activation has pleiotropic effects such as induction of necrosis, mitochondrial damage, proinflammatory actions, and reprogramming of gene expression, that worsen free radical-mediated pathologies [109]. PARP level and activity are also strongly correlated with mitochondrial activity [56]. For example, long-term PARP activation due to depletion of NAD^+^ cell pools lead to a shutdown of mitochondrial function. Conversely, mitochondrial activity is not only maintained upon inhibition of PARP1/2, but further enhanced by activation of SIRT1. This feature has been demonstrated in a number of studies, including the cardioprotective effect in the model of doxorubicin-induced cardiomyopathy [56,59]. In turn, induction of SIRT1 is able to protect against oxidative stress. SIRT1 modifies numerous components of the cell cycle coordination mechanism (e.g., p53 and FOXO) in oxidative damage that results in cell cycle arrest and suppression of apoptosis [110,111]; it induces antioxidant defense systems such as manganese superoxide dismutase (MnSOD), restores mitochondrial biogenesis damaged by oxidative stress [112], and promotes the activation of autophagy, which is impaired by doxorubicin and leads to the accumulation of nondegradable autolysosomes [113].

Therefore, activation of SIRT1 and PARP has opposite characteristics under conditions of oxidative stress because PARP activation contributes to additional damage to cells and tissues during oxidative stress. Thus, the study of the PARP-SIRT interactions will help to understand their functional role/mechanisms in the metabolism of NAD^+^ in order to use them in the future for therapeutic effects against pathologies of the cardiovascular system, as in the development of doxorubicin cardiomypathy.

**Figure 4:**
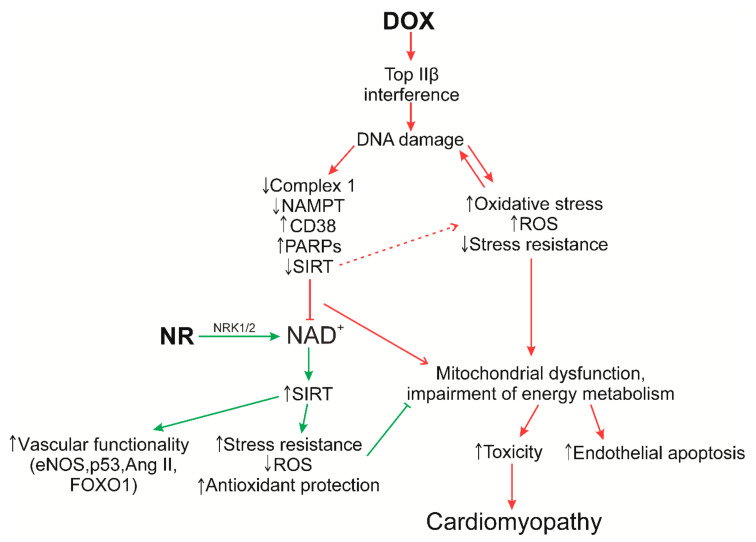
Hypothesis of the impact of NR in the development of doxorubicin cardiomyopathy. It is a popular assumption that the development of doxorubicin (DOX) cardiotoxicity occurs due to the massive generation of ROS, which in turn is caused by a secondary mechanism due to the suppression of topoisomerase 2β. In consequence DNA damage increases, enzymes involved in its repair, PARPs and CD38, are activated, while the activity of sirtuins and NAMPT (the main pathway of NAD^+^ synthesis through NAM under normal physiological conditions, *Core Recycling Pathway*) is suppressed, and the work of Complex I in the electron transport chain is disrupted, using NADH as an electron donor creating NAD^+^. Damage to Complex 1 and ATP production leads to accumulation of ROS that increase oxidative stress. Separately doxorubicin reduces tight junction formation by decreasing the expression of the occluded zone (ZO)^−1^, which can increase doxorubicin levels in the capillary endothelium of the heart muscle. This also reduces nitric oxide (NO) level by enzymatic inhibition, reducing the level of endothelin (ET)^−1^ accumulation of ROS [116]. All mechanisms lead to a significant decrease in NAD^+^ levels, the inability of the antioxidant system of the cardiomyocyte to cope with increased oxidative stress, and mitochondrial dysfunction, which ultimately leads to apoptosis of endothelial and cardiac cells. The administration of nicotinamide riboside (NR) can significantly increase the level of NAD^+^ through a less energy-intensive synthesis via NRK1/2. An increase in the level of NAD^+^ activates the work of sirtuins, which are able to induce antioxidant defense systems that restore mitochondrial biogenesis damaged by oxidative stress and promote the activation of autophagy, which is disturbed by doxorubicin leading to the accumulation of nondegradable autolysosomes. NMN synthesis via NR inhibits endothelial inflammation and improves NO-dependent function. Activation of endothelial SIRT1 controls endothelial homeostasis and vascular functionality by modulating the activity of endothelial nitric oxide synthase (eNOS), p53, angiotensin II receptor (Ang II), FOXO1 and other mechanisms [117].

It is important to note that impaired NAD^+^ homeostasis due to mitochondrial dysfunction is central to the development of cardiac hypertrophy, heart failure, and cardiomyopathy. Changing the redox capacity of the heart further increases its susceptibility to stress. Furthermore, a transition from fatty acid oxidation and oxidative phosphorylation to other forms of substrate metabolism (glycolysis and oxidation of ketones) often occurs with the development of heart failure and cardiomyopathy; while the NAD^+^/NADH ratio decreases, the NAMPT enzyme is repressed [91]. In this regard, the addition of NR is of particular interest, since it is able to normalize the NAD^+^/NADH ratio in the myocardium, exhibits protective effects against unfavorable cardiac remodeling and, importantly, activates the synthesis of NAD^+^ through NRK1/2. There is evidence that the NAMPT enzyme is repressed in some mouse models of cardiac injury, while the expression of NRK2 is greatly increased. A similar shift is observed in humans with cardiomyopathy [114]. Thus, it has been suggested that activation of NAD^+^ synthesis via the NRK2 pathway represents a common adaptive mechanism in heart failure, while the *Nrk2* gene may be activated in response to NAMPT inhibition [30]. Moreover, the synthesis of NAD^+^ via NRK1/2 is a more economical in the consumption of ATP molecules (1 molecule), while the synthesis using NAMPT requires three ATP. An interesting feature of NR is that the activation of SIRT mechanisms and maintenance of Ca^2+^ homeostasis is stimulated by increasing the production of NAD^+^ [115]. In turn, activation of sirtuins is able to protect against cardiac hypertrophy, metabolic dysregulation and cardiac inflammation. These data confirm the preference of NR as a precursor of NAD^+^ in therapeutic use and in maintenance therapy (Figure 4).

Additionally, the question arises about the method of administration of NR to patients undergoing chemotherapy. As indicated in the previous sections, the drug is often administered orally and at the same doses intraperitoneally (not possible in humans). An interesting possibility is the delivery of NR intravenously [108], given that people undergoing chemotherapy receive, experience side-effects that affect the operation of various organ systems, including the digestive tract (intestinal mucositis) [117,118].Therefore, taking the required dose of NR orally may be ineffective due to possible intestinal malabsorption. Direct intravenous administration of the drug could be considered a good alternative in supportive care of the patient.

Considering that the damaging effect of doxorubicin is cumulative and progressive, it is important to consider the frequency of administration of NR to patients undergoing chemotherapy. Studying the characteristics of the time of accumulation and consumption of NAD^+^ during injections of NR, as well as considering the options for its administration regarding the mechanisms of development of anthracycline cardiomyopathy, can play an important role in the prevention or treatment of doxorubicin cardiotoxicity.

The administration of NR shows efficacy in the normalization of several metabolic pathways, such as oxidative stress, inflammatory response, and circadian rhythm. Long-term administration of NR can be a highly effective way to maintain increased SIRT1 activity in tissues and organs where NAMPT-mediated NAD^+^ biosynthesis is impaired, and to resist cardiovascular pathologies that developed during chemotherapy, as well as being a therapy for doxorubicin cardiomyopathy. However, all possible mechanisms of NAD^+^ metabolism and regulation of NAD^+^-mediated proteins are still not clear and require more qualitative study.

## Figures and Tables

**Table 1:** Effects of NR on small laboratory animal models.

No. Reference	Sex, Age	Dose of NR	Duration/Administration Frequency	Route of Administration	Effect
[93]	C57Bl/6J mice, male, 8 weeks	400 mg/kg	12 weeks	diet	Enhanced oxidative metabolism; protection against high fat diet-induced metabolic abnormalities; improved insulin sensitivity
[97]	Tg2576 mice	250 mg/kg	3 months	diet	Benefited cognitive function and synaptic plasticity
[94]	C57Bl/6J mice, male, 8–10 weeks	1000 mg/kg	twice daily for 5 days	IP	Activated a NAD^+^-SIRT3 pathway; reduces neurite degeneration
[77]	unspecified	400 mg/kg	4 weeks	diet	Improved mitochondrial respiratory capacity in muscle
[99]	C57BL/6J mice, male	500 mg/kg	6 days	IP	Improved glucose tolerance; reduced weight gain, liver damage and the development of hepatic steatosis in prediabetic mice; protect against sensory neuropathy
[35]	C57Bl/6J mice, 12-week-old male; 6–8-week-old	185 mg/kg; 500 mg/kg	1 week; 6 days	diet; IP	The increase in NAAD is a highly sensitive biomarker of effective NAD^+^ repletion.
[96]	C57BL/10ScSn-Dmd^mdx^/J mice, male	400 mg/kg	6–8 weeks	diet	Induced the mitochondrial unfolded protein response; delayed senescence of neural SCs and melanocyte SCs; increased mouse life span
[101]	C57BL/6 mice, male, 2 months	100, 300, 500 mg/kg	single dose	IP	prevented lung and heart injury; improved the survival in sepsis
[102]	C57BL/6 mice, male, 2 months	100, 300, 500 mg/kg	single dose	IP	Elevated NAD^+^ levels, reduced cardiac injury and myocardial dysfunction
[103]	BALB/c mice, male, 3 months	1000 mg/kg	single dose	IP	Protective effects of NR treatment in a mouse model of retinal degeneration
[104]	Sprague-Dawley rats, male and female	5000 mg/kg; 750, 1500, 2500, 5000 mg/kg;, 300, 1000, 3000 mg/kg	Single; 14 days; 90 days	gavage	Toxicity profile similar to nicotinamide, target organs of toxicity were liver, kidney, ovaries, and testes; the lowest observed adverse effect level for NR was 1000 mg/kg/day; the no observed adverse effect level was 300 mg/kg/day
[105]	Wistar rats, male, 4 months	300 mg/kg	21 days	gavage	Negative effect of NR administration on physical performance
[106]	Wistar rats, male, 4 months	300 mg/kg	21 days	gavage	Increased NADPH levels in liver, but not in muscle, decreased the activity of major antioxidant enzymes in muscle; excessively increased glycogen in liver, but not in muscle; decreased glucose concentrations in blood; decrease maximal lactate production during exercise
[107]	Sprague-Dawley rats, female	200 mg/kg	7 days prior to and 24 days post-paclitaxe; 21 days beginning 14 days post-paclitaxel	gavage	Reversed the well-established tactile hypersensitivity in a subset of rats and blunte escape–avoidance behavior
[108]	Wistar rats, male	50 mg/kg	single dose	IV	Protect the intestinal wall from ischaemia-reperfusion injury; improving the relaxation function of mesenteric vessels
[57]	Wistar rats, male	400 mg/kg	28 days	IP	Reduced adiposity (visceral and subcutaneous); improved insulin resistance; increased the antioxidant capacity via glutathione peroxidase and catalase enzymes (in rats under calorie restriction)

NR, nicotinamide riboside; IP, intraperitoneal; IV, intravenous.
